# The COL11A1/Akt/CREB signaling axis enables mitochondrial-mediated apoptotic evasion to promote chemoresistance in pancreatic cancer cells through modulating BAX/BCL-2 function

**DOI:** 10.7150/jca.47032

**Published:** 2021-01-01

**Authors:** Hui Wang, Runling Ren, Zizhong Yang, Jun Cai, Shaoxia Du, Xiaohong Shen

**Affiliations:** School of Medicine, Nankai University, Tianjin 300071, China.

**Keywords:** COL11A1, Akt, GEM, apoptosis, chemoresistance

## Abstract

Collagen XI, a member of the collagen family, is present in the extracellular matrix (ECM), and high collagen XI/αI (COL11A1) expression in tumor tissue is reportedly correlated with the clinicopathological parameters of pancreatic ductal adenocarcinoma (PDAC). However, the function of COL11A1 in the development of pancreatic cancer cells remains unclear. In the current study, we assessed mRNA expression of COL11A1 and its receptors and created a testing-model of both a COL11A1-overexpressing tumor microenvironment and/or altered-COL11A1 expression in pancreatic cancer cell lines. Next, we investigated the mechanism by which COL11A1 affects growth, gemcitabine (GEM) resistance and apoptosis in pancreatic cancer cells. We demonstrated that COL11A1 phosphorylated Akt^Ser473^, promoting proliferation of cancer cells and inhibiting their apoptosis. Additionally, our data showed that COL11A1/Akt/CREB altered the balance between BCL-2 and BAX and mediated their mitochondrial translocation in pancreatic cancer cells. The COL11A1/Akt axis induced disruption of mitochondrial transmembrane function, enabling mitochondria-mediated apoptotic evasion to promote chemoresistance. We also explored the regulatory effect of COL11A1/Akt on molecular signaling in the mitochondria-mediated apoptotic program. COL11A1/Akt disturbed the BCL-2/BAX balance, inhibiting cytochrome c (Cyt-C) release and binding of Apaf-1/procaspase-9/Cyt-C, which suppressed the apoptotic program and induced GEM resistance in pancreatic cancer cells. In conclusion, COL11A1 modulates apoptotic inhibition and chemoresistance in pancreatic cancer cells by activating the Akt/CREB/BCL-2/BAX signaling pathway. COL11A1 may represent a distinct prognostic indicator and may be an attractive therapeutic target for PDAC.

## Introduction

Pancreatic ductal adenocarcinoma (PDAC), one of the most malignant neoplasms, is devastating and has an extremely poor prognosis 1-3. The pattern of tumor development in this cancer, with its insidious onset, rapid progression and chemotherapeutic resistance, contributes to the intractability of PDAC. Several factors are involved in the aggressive nature of PDAC, but the mechanisms whereby they interact are still under investigation. Several studies have suggested that cancer cell attachment to the ECM evokes biomolecular activity, which generates an ECM microenvironment appropriate for cancer cell growth and pancreatic cancer survival. In addition, a large number of differentially expressed proteins involved in tumor growth, migration, angiogenesis, and metastasis have been identified in the ECM of PDAC 4, 5. Notably, an investigation of ECM-specific proteins might reveal the basis for new diagnostic tools and treatments in PDAC.

The tumor ECM is usually composed of collagens, fibronectins, laminins, glycosaminoglycan and proteoglycan. The desmoplastic response is a predominant feature of pancreatic cancer, unlike most other tumors, and contributes to disease progression. Both mesenchymal cells and cancer cells themselves secrete large amounts of ECM proteins into the tumor microenvironment 6-10. The ECM in PDAC is surrounded by extensive quantities of interspersed fibrillar, collagen-rich stroma 10, 11. Recently, a proteomics analysis of the ECM in PDAC indicated that collagens derived from cancer cells in the microenvironment represent a critical factor influencing overall survival 12. Consequently, cooperation between tumors and collagen is pivotal for PDAC progression. To date, 42 different collagen genes encoding 28 different types of collagens have been identified. Collagens are trimeric molecules consisting of three polypeptide α-chains that include the repeated sequence (G-X-Y)n, in which X is frequently proline and Y is hydroxyproline; these α-chains form a triple helix. Fibril-forming collagens include collagen I, II, III, V, XI, XXIV and XXVII. Each of these collagens contains at least one triple-helical domain and is secreted and deposited into the ECM 13, 14. Fibrillar collagen is synthesized as procollagen. Once secreted to the extracellular milieu, procollagen is proteolytically cleaved and converted to mature collagen, which assembles in the ECM 15. Collagen types I, II, III and V are upregulated in PDAC and other malignant tumors, where they influence tumor development by regulating molecular cascades 7, 16-18. However, the specific mechanisms of collagens and their molecular manipulation of tumorigenesis in PDAC require further investigation. Collagen XI, which was first identified in cartilage, is expressed at low levels in normal tissue but is highly expressed in malignant tissue, making it a candidate for a prominent marker with which to investigate neoplastic pathogenesis. Recent studies have suggested the tumorigenic property of stromal COL11A1 overexpression in various carcinomas 19-23. Additionally, high tissue (both epithelial cells and stroma) COL11A1 expression is reportedly correlated with the clinicopathological parameters of PDAC 24-26. Thus, COL11A1 might be a promising molecule for innovative research on anticancer strategies in PDAC.

Cell-collagen interactions mediate the biological activity of cancer cells. Collagen-specific receptors called integrins (integrin α1β1, α2β1, α10β1, and α11β1) and discoidin domain receptors (DDRs) have been extensively studied. Integrin α1β1/α2β1 and DDR1/2 exert distinct functions in cell-collagen coactivity. In particular, integrin α1β1 and DDR2 were reported to activate Akt for biomolecular signal cascade transduction during various oncogenic processes 27-29. Akt/PKB functions as a critical regulator of cell growth, proliferation and survival. Full activation of Akt/PKB requires its phosphorylation at a second site, Ser473. This phosphorylation has direct effects on the apoptosis pathway, targeting BCL-2-related apoptotic proteins and affecting their transcription and activity in response to apoptotic stimuli 30. Many molecular events are involved in initiating cancer development. Among them, apoptosis evasion is a hallmark of cancer, and inhibition of apoptotic signaling facilitates oncogenesis 31. There are two distinct cellular apoptosis pathways: the intrinsic (or mitochondrial) and extrinsic (or death receptor) pathways. The intrinsic mitochondrial pathway has been proposed as remarkably linked to cancer development 32. The BCL-2 family regulates cell mitochondrial apoptosis. The antiapoptotic proteins BCL-2, BCL-XL, MCL-1, BCL-W, and BFL-1/A1 interrupt the functions of the proapoptotic effectors BAX and BAK, preventing apoptosis. Therefore, COL11A1/integrinα1β1/DDR2 signaling to inhibit the apoptotic process through PI3K/Akt has been demonstrated in other cancer types 33-35. Furthermore, most approved chemotherapies kill cancer cells by switching on the apoptotic program. However, chemoresistance poses a persistent problem in cancer treatment. Therefore, it is important to determine the specific molecules that respond to apoptotic priming to identify united therapeutic regimens that show improved sensitivity compared to conventional chemotherapy 36. GEM is a nucleoside analog used as a standard chemotherapeutic agent for PDAC treatment. GEM is a cell cycle-specific agent, and its phosphorylation forms GEM triphosphate (dFdCTP) in the nucleus. dFdCTP inhibits DNA polymerase to mediate DNA double-strand breaks, which induce apoptosis, comprising the mechanism by which GEM is toxic to cancer cells. GEM resistance acquired by individual patients during treatment cycles remains a major clinical challenge. The intrinsic mechanisms related to chemoresistance are complex and include transporter dysfunction, DNA repair and apoptotic resistance 37-39. Among the underlying causes of chemoresistance, apoptotic depletion might be the most critical. Consequently, further investigation is needed.

In this study, we suggest that COL11A1/Akt modulates apoptotic inhibition and induces chemoresistance in pancreatic cancer cells. We assessed the mechanisms related to the functions of COL11A1/Akt signaling in regulating cancer cell apoptosis. We showed that COL11A1 activates Akt/CREB signaling, resulting in BCL-2-related apoptotic protein transformation, which induces mitochondrial dysfunction, blocks the apoptotic program and enhances chemoresistance of PDAC. We propose that COL11A1/Akt represents a novel target for therapeutic development in pancreatic cancer.

## Materials and Methods

### Cell culture and transfection

Pancreatic cancer cell lines were purchased from the Cancer Institute & Hospital of the Chinese Academy of Medical Sciences (Shanghai, China). PANC-1 and Mia PaCa-2 cells were cultured in RPMI-DMEM (Biological Industries, Kibbutz Beit-Haemek, Israel) with 10% serum (BI, Australia). Capan-1 and BxPC-3 cells were grown in RPMI-1640 medium supplemented with 10% serum. All cells were cultured in a humidified incubator at 37 °C with 5% CO_2_. We purchased small interfering RNAs (siRNAs) targeting COL11A1, integrin α1β1 and DDR2 from Synbio Tech. The siRNA sequences for target genes are listed in Table 1. The INTERFERin® transfection reagent (Polyplus, France) was used to transfect cells with siRNAs. Cells were starved of serum for 24 h.

### ELISA evaluation

Cells were plated in 6-well plates and incubated at 37 °C for 24 h. Subsequently, we measured COL11A1 content in the medium using a human COL11A1 ELISA kit (NOVUS, #NBP2-75829, US). The samples and standards were loaded onto the bottom of a microELISA strip plate without touching the well wall. The microELISA strip plate was incubated at 37 °C for 30 min after being sealed with a membrane. Then, the membrane closing the plate was carefully peeled off, the solution in the plate was aspirated, and the plate was filled with wash solution. The wash solution was discarded after a 30 s incubation. The washing procedure was repeated 5 times. Then, 50 μl HRP-conjugated reagent was added to every well except for the blank control well, and the plate was sealed with a membrane and incubated for 30 min at 37 °C. The washing procedure was repeated 5 times. Then, 50 μl chromogen solution A and 50 μl chromogen solution B were added to each well, and the plate was mixed with gentle shaking followed by incubation for 15 min at 37 °C in the dark. A stop solution was added to terminate the reaction. The color of the well changed from blue to yellow. We read OD values at 450 nm using a microtiter plate reader. The OD value of the blank control well was set as zero. The measurement was performed within 15 min of adding the stop solution.

### Western blotting

Cell lines were solubilized with lysis buffer (Sangon Biotech, Shanghai, China). Cell culture supernatants were collected and concentrated 20-fold by Amicon Ultra 4 centrifugation (UFC0096, Millipore, Billerica, MA) 40. Following centrifugation and quantification, an equal volume of 1× loading buffer was added to the protein samples, which were then denatured at 100 °C for 10 min. Then, equal amounts of protein (30 μg) from each sample were loaded on a 10% gel for SDS-PAGE, after which they were transferred onto PVDF membranes (Bio-Rad). The membranes were then blocked with 5% nonfat milk for 1 h at room temperature and probed with the following antibodies at 4°C overnight as appropriate: anti-COL11A1 (1:1,000, ab64883, Abcam, UK), anti-Akt (1:1,000, Proteintech, USA), anti-p-AktSer473 (1:1,000, Cell Signaling Technology, USA), anti-Erk1/2 (1:2,000, Proteintech, USA), anti-p-Erk1/2 (1:2,000, Cell Signaling Technology, USA), anti-Jnk (1:200, Santa Cruz Biotech, USA), anti-p-Jnk (1:200, Santa Cruz Biotech, USA), anti-BCL-2 (1:2,000, Cell Signaling Technology, USA), anti-caspase-9 (1:1,000, Abcam, UK), anti-BAX (1:1,000, Proteintech, USA), anti-caspase-3 (1:1,000, Abcam, UK), anti-β-actin (1:10,000), anti-p-BAX^Ser184^ (1:2,000, Abcam, UK) anti-p-CREB^Ser133^ (1:2,000, Abcam, UK), anti-CREB (1:2,000, Abcam, UK), anti-ANT (1:2,000, Abcam, UK), and anti-β-actin (1:10000, ZSGB-BIO, China). After incubation with corresponding secondary antibodies, proteins were detected using ECL (Millipore, USA). ImageJ software was used to quantify western blotting bands.

### RT-qPCR detection

Total RNA was obtained using an Eastep total RNA extraction kit (Promega, Wisconsin, WI, USA). Total RNA from each sample was then subjected to reverse transcription using GoScript^TM^ reverse transcriptase (Promega, Wisconsin, WI, USA). RT-qPCR was performed with GoTaq® qPCR Master Mix (Promega, USA) in a 20 μl reaction volume, and detection was performed using a DA7600 real-time nucleic acid amplification fluorescence detection system (Bio-Rad). The 2^-ΔΔCt^ method was used to determine relative expression levels. We quantified GAPDH mRNA levels as an internal quantity control. All primers used for RT-qPCR were obtained from Sangon Biotech (Shanghai, China). RT-qPCR products were then subjected to electrophoresis. The primer sequences of target genes are listed in **Table 2.**

### COL11A1 coating

We purchased COL11A1 from ImmunoClone (#IC8157, USA). COL11A1 was added to the wells of a plate and allowed to absorb overnight at 4 °C. Then, the wells were washed with PBS to remove unbound proteins and blocked with 0.2% BSA (Millipore) for 30 min at 37 °C. Finally, the wells were washed twice with sterile PBS and sterilized by UV exposure for 30 min. COL11A1 at 1 µg/ml was used to coat the wells in plates used to assess cell apoptosis and GEM resistance.

### Cell viability measurement

Cells were seeded in 24-well plates in DMEM/RPMI-1640 complete medium and allowed to attach to the well overnight before application of different treatments. After 24 h of treatment, cells were seeded in 96-well plates. After 24 h, 48 h and 72 h, cells were counted using a Cell Counting Kit-8 (Dojindo, Japan) assay following the manufacturer's protocol, and absorbance was measured at 450 nm. The CCK-8 assay was used to test the cytotoxicity of GEM (20 μM). Cell viability was measured using OD values at 450 nm. The survival rate was calculated with the formula [(As-Ab)/(Ac-Ab)]×100%, where As represents the absorbance of experimental groups, Ab represents the absorbance of the blank sample containing complete medium without cells and Ac represents the control group containing cells with no treatments. The IC_50_ was calculated by nonlinear least squares curve fitting.

### Flow cytometry analysis

BxPC-3 cells were seeded in 12-well plates. Cells were plated in COL11A1-coated plates for 24 h and then treated with GEM or staved for 24 h or 48 h. Following different treatments, cells were collected, washed twice with cold PBS and stained with PI and FITC Annexin-V for 15 min (Apoptosis Detection Kit, Keygen, China). Then, cells were immediately analyzed by flow cytometry. The percentage of the cell population in apoptosis was calculated using FlowJo software.

### Confocal microscopy assays

BxPC-3 cells were plated onto 24-well plates and incubated for 24 h at 37 °C. Then, different treatments were performed. After 24 h, cells were resuspended and placed on coverslips in a 24-well plate. Transfected cells were incubated first with MitoTracker Red CMXRos (200 nM) (CST #9082) in complete medium at 37 °C for 30 min. The cells were fixed for 15 min in 4% formaldehyde at room temperature and permeabilized with Triton X-100 (0.1%). Then, cells were blocked with 20% goat serum for 1 h and incubated at room temperature with anti-BAX, anti-BCL-2 and anti- Cyt-C antibodies (diluted 1:200) for 1 h. Cells were then probed with the corresponding secondary antibodies for 1 h at room temperature. Finally, cells were incubated with DAPI (1:5,000) for 10 min and washed three times with PBST, and the coverslips were placed onto microscope slides. A laser scanning microscope was used to obtain the resulting images.

### Mitochondrial transmembrane potential (Δψ_m_) assessment

Cell Δψ_m_ was detected by flow cytometry and immunofluorescence. For immunofluorescence experiments, BxPC-3 cells were seeded in 24-well plates and incubated for 24 h at 37 °C. Then, different treatments were performed. After 24 h, cells were resuspended and placed on coverslips in a 24-well plate. Transfected cells were first incubated with JC-1 (10 μg/ml) (Solarbio #J8030) in complete medium at 37 °C for 15 min. Cells were then fixed for 20 min in 4% formaldehyde at room temperature. Finally, cells were incubated with DAPI (1:5,000) for 10 min and washed three times with PBST, and the coverslips were placed onto microscope slides. A laser scanning microscope was used to obtain images. For flow cytometry experiments, BxPC-3 cells were seeded in 12-well plates. Following different treatments, cells were stained with JC-1 (10 μg/ml) for 15 min, collected, and washed twice with cold PBS. Then, cells were washed with PBS and analyzed immediately by flow cytometry. The percentage of the cell population in apoptosis was calculated using FlowJo software.

### Coimmunoprecipitation analysis

Cell lysates were treated with protein A/G agarose beads (Millipore) at 4 °C for 1 h to reduce nonspecific binding. Then, lysates were incubated with the indicated antibodies, either anti-BAX antibody (Proteintech) or anti-Apaf-1 antibody (Abcam, USA). Following incubation at 4 °C overnight, the lysates were then probed with protein A/G agarose beads for another 2 h. The immunoprecipitated complexes were subjected to western blotting and probed with anti-BCL-2, anti-BAX, anti-VDAC, anti-ANT and anti-procaspase-9 antibodies.

### ChIP assay

Native protein-DNA complexes were cross-linked by treatment with 1% formaldehyde for 15 min, and ChIP assays were performed as previously reported 41. Briefly, equal aliquots of isolated chromatin were subjected to immunoprecipitation with anti-CREB and IgG monoclonal antibodies.

### Statistical analysis

Data from at least 3 independent experiments are presented as the mean ± standard deviation (SD). Student's t-test and ANOVA were used for comparisons between experimental groups and the control group. Differences for which *p* < 0.05 were considered statistically significant.

## Results

### COL11A1/α1β1/DDR2 facilitates growth of pancreatic cancer cells and their resistance to GEM

**Figure 1:**
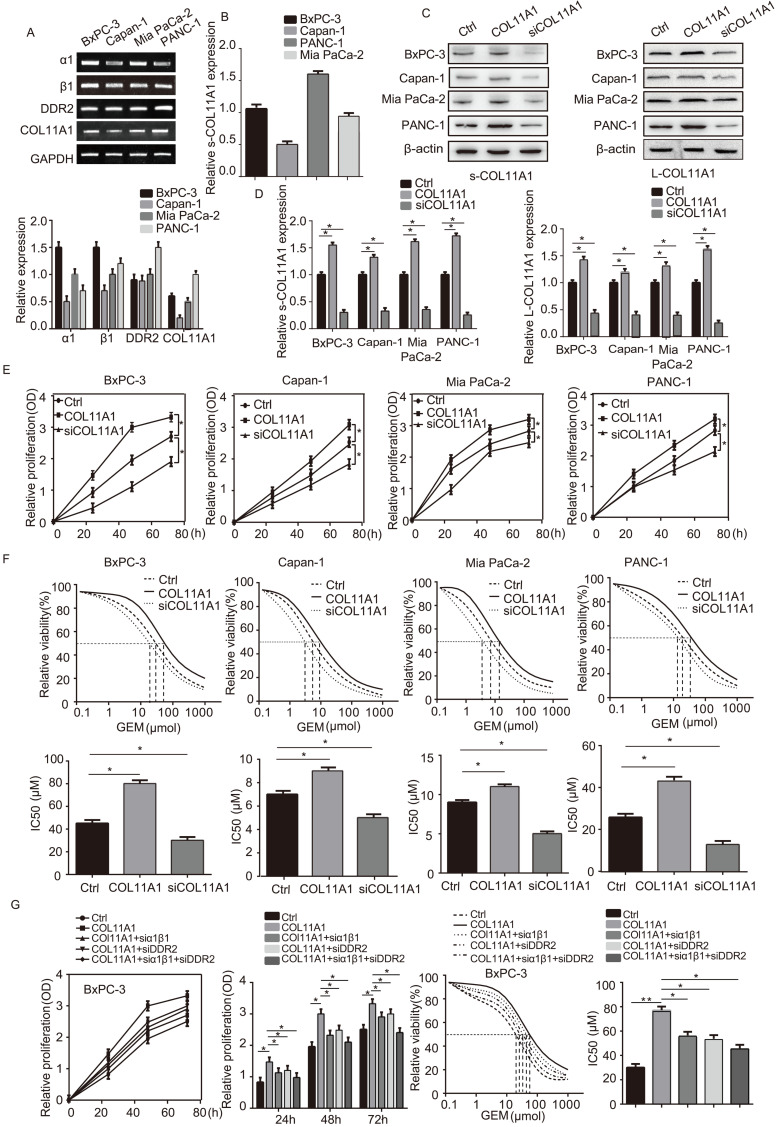
** COL11A1 promoted the proliferation of pancreatic cancer cells and the resistance to GEM via integrin α1β1/DDR2 receptors.** (A) The mRNA expression of COL11A1, integrin α1β1 and DDR2 were assessed by RT-qPCR in pancreatic cancer cells (BxPC-3, Capan-1, Mia PaCa-2 and PANC-1). (B) The expression of COL11A1 in cell culture supernatant (s-COL11A1) protein expression levels were assessed by ELISA in BxPC-3, Capan-1, Mia PaCa-2 and PANC-1 cells. (C) s-COL11A1 and L-COL11A1 (the levels of COL11A1 in total cells lysates) of the four pancreatic cancer cell lines (BxPC-3, Capan-1, Mia PaCa-2 and PANC-1) were detected after stimulation with different treatments by western blotting. (D) The quantitative chart of s-COL11A1 and L-COL11A1 protien expression level. (E) BxPC-3, Capan-1, Mia PaCa-2 and PANC-1 cells were treated with COL11A1 (1 µg/ml) or siCOL11A1, and cell proliferation was determined by CCK-8 assay. (F) The IC50 and cytotoxic effects of GEM (20 µM) on BxPC-3, Capan-1, Mia PaCa-2 and PANC-1 cells treated with COL11A1 or siCOL11A1 were evaluated by CCK-8 assay. (G) Cell proliferation, the IC50 and the cytotoxic effects of GEM in BxPC-3 cell following different treatments were determined by CCK-8 assay (n=3, *p<0.05).

We assessed mRNA levels of COL11A1 and its receptors (integrin α1β1 and DDR2) by RT-qPCR in the pancreatic cancer cell lines BxPC-3, Capan-1, Mia PaCa-2 and PANC-1 (Fig. 1A). Next, expression of COL11A1 in cell culture supernatants (s-COL11A1) was tested in BxPC-3, Capan-1, Mia PaCa-2 and PANC-1 cell lines by ELISA (Fig. 1B). In addition, s-COL11A1 and the levels of COL11A1 in pancreatic cancer total cell lysate (L-COL11A1) in four pancreatic cancer cell lines was detected after stimulation with different conditions by western blotting. Results revealed that COL11A1-coated increased both the expression level of s-COL11A1 and L-COL11A1 in pancreatic cancer cells, and siCOL11A1 markedly reduced expression of COL11A1 in cancer cells (Fig. 1C,D). To further confirm this, we detected the expression level of s-COL11A1 in supernatant medium and L-COL11A1 in total cell lysate of the four pancreatic cancer cells (BxPC-3, Capan-1, Mia PaCa-2, PANC-1) treated with COL11A1 protein for 0h, 12h, 24h, 48h by western blotting. The results showed that COL11A1-coated could promote the expression of s-COL11A1 and L-COL11A1 in a time-dependent manner. And, the expression level of s-COL11A1 was tested by ELISA in the four pancreatic cancer cells (BxPC-3, Capan-1, Mia PaCa-2, PANC-1) treated with coating-COL11A1 for 48 h. The result of ELISA is consistent with western blotting (Fig. S1A,B). Therefore, the fragment of COL11A1 promoted the expression of the full-length COL11A1.To investigate whether COL11A1 affects the proliferation of pancreatic cancer cells and their resistance to GEM, we increased and knocked down COL11A1 in cancer cells using COL11A1-coated plates and treatment with siRNA against COL11A1, respectively, with or without GEM treatment. Both proliferation and GEM resistance were markedly enhanced in the cells grown in COL11A1-coated plates compared to the control group of pancreatic cancer cells, especially in the BxPC-3 cell line. However, after COL11A1 was knocked down in pancreatic cancer cells by siRNA, cell proliferation and GEM resistance were decreased (Fig. 1E and F). Next, we used BxPC-3 cells to determine the effect of the receptors on proliferation and GEM resistance. When cells were individually or simultaneously transfected with siRNAs against integrin α1β1 and DDR2, the function of COL11A1 in enhancing cell proliferation and GEM resistance was attenuated. Additionally, the effect of simultaneous knockdown of integrin α1β1 and DDR2 with siRNAs was more pronounced than that of individual knockdown (Fig. 1G). Therefore, these results indicate that the COL11A1/integrin α1β1/DDR2 axis promotes proliferation and GEM resistance in pancreatic cancer cells, and integrin α1β1/DDR2 receptors mediate the function of COL11A1 in facilitating cell growth.

### The COL11A1/α1β1/DDR2/Akt axis regulates the function of BCL-2/BAX and suppresses apoptosis in pancreatic cancer cells

**Figure 2:**
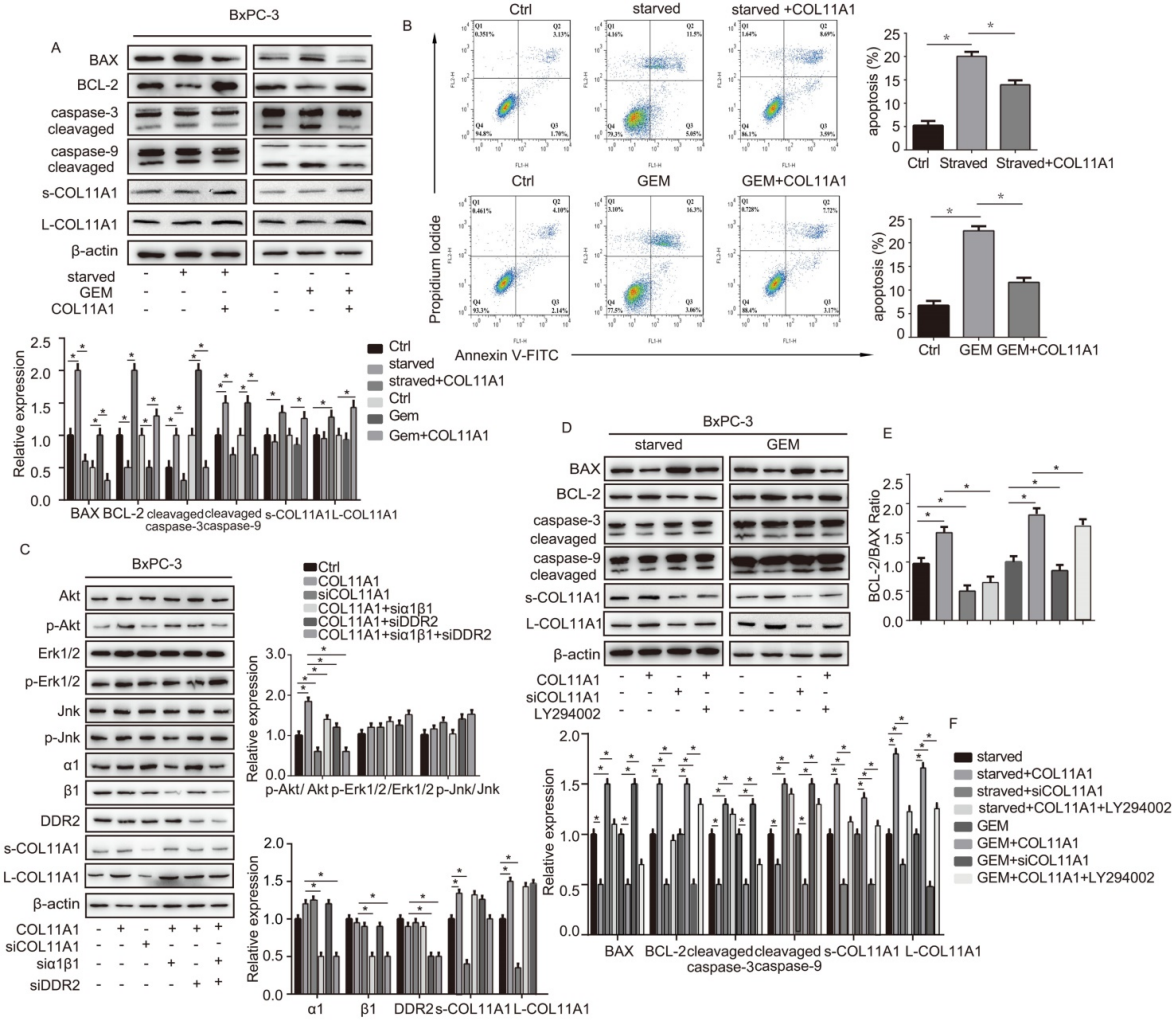
** The COL11A1/α1β1/DDR2/Akt axis affects apoptosis-related protein function and cancer cell apoptosis.** (A) Expression levels of the s-COL11A1, L-COL11A1, BCL-2, BAX, caspase-3/cleaved caspase-3 and caspase-9/cleaved caspase-9 proteins in BxPC-3 cell under serum starvation or treated with GEM (20 µM) and COL11A1 (1 µg/ml) were examined by western blotting. (B) BxPC-3 cell treated with serum starvation or GEM and COL11A1 were stained with PI and FITC Annexin-V and analyzed by flow cytometry. (C) Western blotting was used to determine the protein expression levels of s-COL11A1, L-COL11A1, α1β1, DDR2, Akt, Erk1/2, Jnk, p-Akt^Ser473^, p-Erk1/2 and p-Jnk after stimulation with different treatments. The relative ratios of p-Akt^Ser473^/Akt, p-Erk1/2/Erk1/2 and p-Jnk/Jnk expression were shown. The chart shows changes in protein expression. (D) Western blotting analysis of s-COL11A1, L-COL11A1, BCL-2, BAX, caspase-3/cleaved caspase-3 and caspase-9/cleaved caspase-9 protein expression in BxPC-3 cell treated with serum starvation or GEM, COL11A1, siCOL11A1 and/or LY294002. (E) The BCL-2/BAX ratios in BxPC-3 cell after different treatments were determined. (F) Legend for the histograms in (D) and (E) (n=3, *p<0.05).

**Figure 3:**
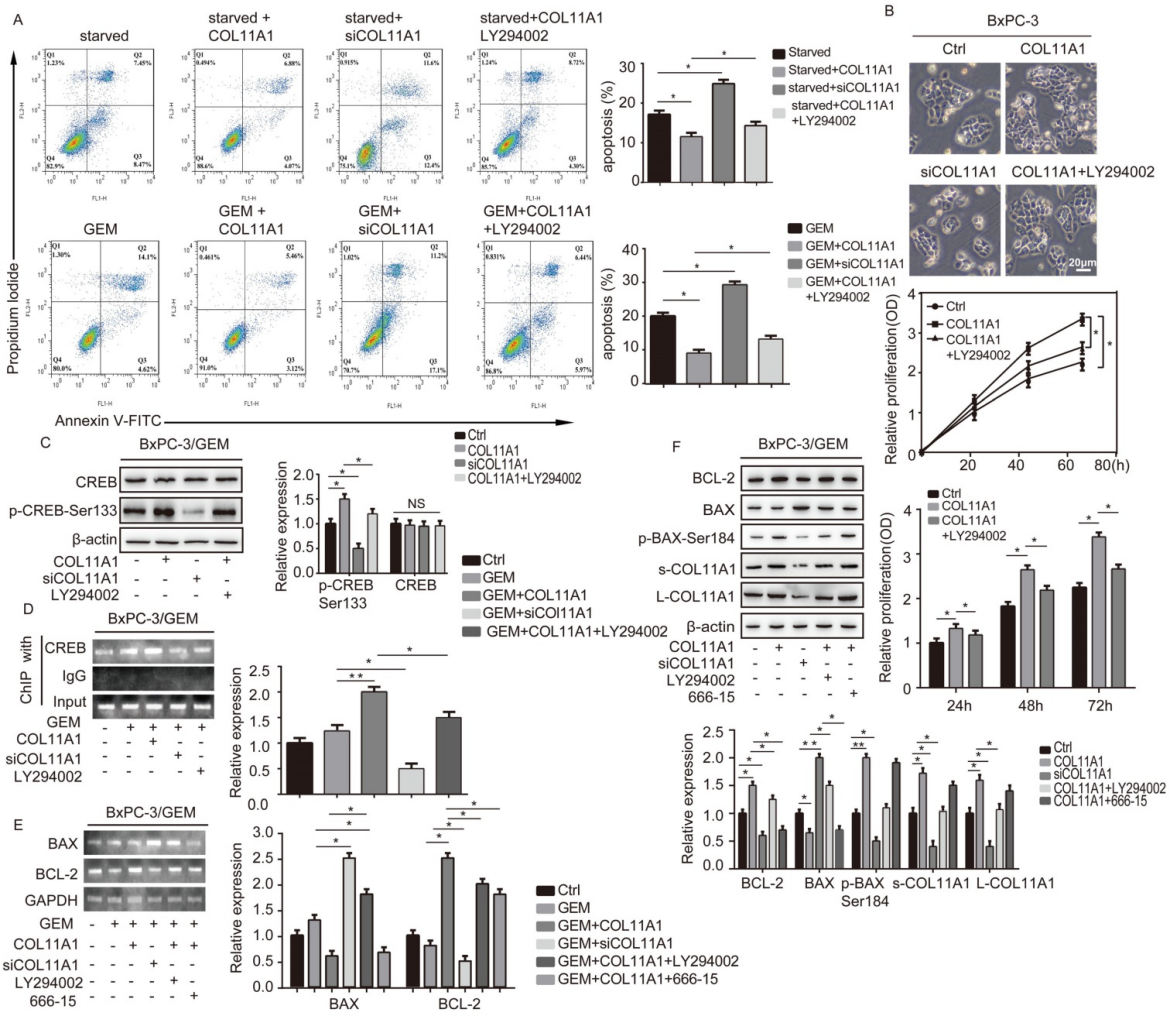
** COL11A1/Akt/CREB signaling disturbed the balance between BCL-2/BAX and mediated GEM-induced apoptosis inhibition.** (A) Flow cytometry cell apoptosis analyses of BxPC-3 cell treated with serum starvation or GEM and COL11A1, siCOL11A1 or COL11A1+LY294002 were carried out. (B) BxPC-3 cell proliferation was detected by photography and quantified, and the CCK-8 assay was carried out. (C) Western blotting was performed to determine the expression of CREB and p-CREB^Ser133^ in BxPC-3 cell after the indicated treatment. (D) CREB ChIP for the promoter of BCL-2. BxPC-3 cell were treated with GEM and COL11A1, siCOL11A1 or COL11A1+LY294002. The product of PCR amplification from total chromatin was used as a positive control, anti-IgG served as a negative control, and anti-CREB showed the interaction between CREB and the BCL-2 promoter after the indicated treatment. (E) The mRNA levels of BCL-2 and BAX in BxPC-3 cell under different conditions were assessed by RT-qPCR. (F) The protein expression levels of s-COL11A1, L-COL11A1, BAX, p-BAX^Ser184^ and BCL-2 in BxPC-3 cell after the indicated treatment was analyzed by western blotting. Data represent the mean ± SD (n=3, *p<0.05).

We evaluated the effect of addition of COL11A1 on apoptosis in starved or GEM-treated BxPC-3 cells using western blotting and flow cytometry. Western blotting results showed that starvation or GEM treatment reduced BCL-2 expression, while increased BAX and cleaved caspase-3/9 expression when compared with the control group (Fig. 2A). However, when starved/GEM-treated cells were treated with COL11A1, BCL-2 expression was increased, and expression of BAX and cleaved caspase-3/9 was reduced compared to the group under starvation or treated with GEM alone (Fig. 2A). GEM treatment induced no significant change in the expression levels of L-COL11A1 and s-COL11A1 (Fig. 2A, S1C), as well as α1β1 and DDR2 (Fig. S1C). The flow cytometry results showed that COL11A1 attenuated the enhanced percentage of apoptotic cells caused by serum starvation or GEM treatment (Fig. 2B). It has been reported that the activity of Akt, Erk1/2 and Jnk signaling molecules is linked with apoptotic and gemcitabine resistance in pancreatic cancer cells 42-48. To further study the mechanism by which COL11A1 affects proliferation and apoptosis in pancreatic cancer cells, we investigated the effect of COL11A1 treatment through coated cell culture plates on activation of the Akt, Jnk and Erk1/2 molecules in BxPC-3 cells. Western blotting showed that COL11A1 significantly increased levels of phosphorylated Akt^Ser473^ compared to the control group, but no obvious changes in expression of p-Erk1/2 and p-Jnk were observed. However, when cells were treated with siRNAs against COL11A1, integrin α1β1 and DDR2, the level of phosphorylated Akt^Ser473^ was significantly reduced, while the expression of integrin α1β1 and DDR2 was not significantly affected, except for the introduction of siRNAs against integrin α1β1 and DDR2 (Fig. 2C). We also detected the expression level of COL11A1, integrin α1β1 and DDR2 in cancer cell total lysates by western blotting to assess the knockdown efficiency of si-COL11A1, siα1β1 and siDDR2 (Fig.S1D). Previous studies have demonstrated that p-Akt^Ser473^ inhibits the activity of P73 (a transcription factor of BAX) by restraining YAP expression 49 and enhancing BCL-2 expression 30. Therefore, we examined the expression of apoptosis-related molecules BCL-2, BAX and caspase-3/9 in cells treated with GEM and COL11A1, siCOL11A1 and/or LY294002 (a PI3K inhibitor). Results showed that COL11A1 increased BCL-2 expression and decreased expression of BAX and cleaved-caspase-3/9 proteins. However, siCOL11A1 exerted the opposite effect, and LY294002 rescued the effects of COL11A1 on apoptotic protein expression. In addition, LY294002 rescued the increase in the levels of s-COL11A1 and L-COL11A1 induced by COL11A1-coated treatment (Fig. 2D). Thus, we sepaculated that the fragment of COL11A1 activated Akt signaling, in turn promoted expression of the full length COL11A1. BCL-2/BAX ratio was increased upon addition of COL11A1 and decreased after treatment with siCOL11A1 or LY294002 (Fig. 2E). In addition, flow cytometry results were similar to those shown in Fig. 2D. COL11A1 reduced the percentage of apoptotic cells, and siCOL11A1 and LY294002 increased the percentage of apoptotic cells (Fig. 3A). Moreover, we discovered that siCOL11A1 and LY294002 inhibited cell proliferation (Fig. 3B). These results demonstrate that COL11A1 promotes cell proliferation and inhibits cell apoptosis by activating Akt in pancreatic cancer cells.

### The COL11A1/Akt/CREB axis disrupts the balance between BCL-2 and BAX and mediates mitochondrial translocation of BCL-2/BAX

**Figure 4:**
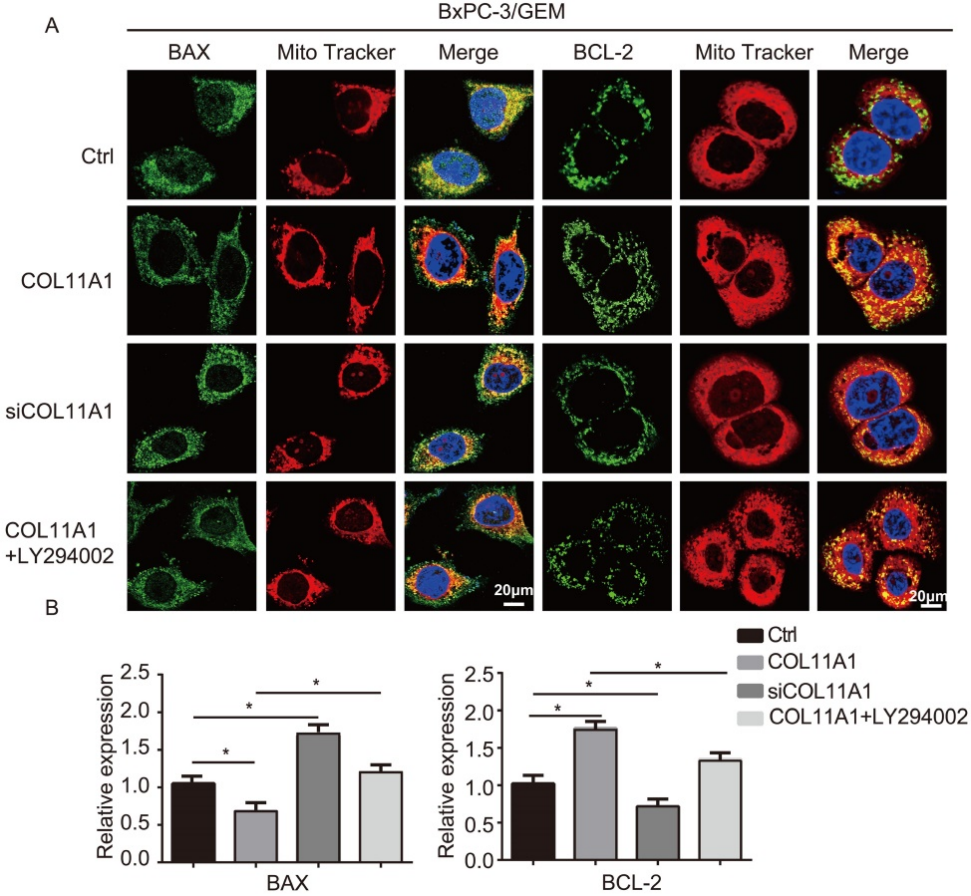
** COL11A1/Akt signaling regulates the mitochondrial translocation of expressed BCL-2/BAX.** (A) The results of immunofluorescence analysis of BAX and BCL-2 expression in BxPC-3 cell following different treatments are shown. Mitochondria were visualized with MitoTracker (red); BAX and BCL-2 were visualized with Alexa Fluor 488 (green), and nuclei were stained with DAPI. Confocal images were taken using a 200× objective. (B) The fluorescence intensity was determined and normalized against the cellular background fluorescence. The levels of BAX and BCL-2 were calculated as a ratio and compared to those in the controls. Data represent the mean ± SD (n=3, *p<0.05).

BCL-2 and BAX play a vital role in implementing the apoptotic process; therefore, we further examined the molecular mechanisms by which COL11A1 mediates its inhibitory activity against apoptosis. Studies have shown that p-Akt^Ser473^ promotes the phosphorylation of CREB at Ser133 50. Therefore, we detected the effect of COL11A1 on p-CREB^Ser133^ levels by western blotting, which demonstrated that COL11A1 increases phosphorylation of CREB; however, siCOL11A1 and LY294002 reduced expression of p-CREB^Ser133^ (Fig. 3C). Therefore, COL11A1 promotes phosphorylation of CREB by activating Akt in BxPC-3 cells, and CREB binds the BCL-2 promoter (-1546 GTGACGTTA -1537) 51, 52. Therefore, we examined the binding activity of CREB and BCL-2 in BxPC-3 cells after their treatment with GEM and COL11A1, siCOL11A1 and/or LY294002. A ChIP assay confirmed that COL11A1 increased the binding of CREB to the BCL-2 promoter, while siCOL11A1 and LY294002 reduced this binding activity (Fig. 3D). Next, after cells were subjected to different treatments, we further detected the mRNA and protein levels of BCL-2 and BAX by RT-qPCR and western blotting, respectively. Results revealed that COL11A1 promotes mRNA expression of BCL-2, while siCOL11A1, LY294002 and/or 666-15 (an inhibitor of CREB) inhibited mRNA expression of BCL-2. mRNA expression of BAX was inhibited by COL11A1 and promoted by siCOL11A1 and LY294002, but 666-15 had no effect on BAX mRNA expression (Fig. 3E). In addition, western blotting results were consistent with RT-qPCR results (Fig. 3F). Moreover, we found that COL11A1 increased BAX phosphorylation at Ser184 by activating Akt and that siCOL11A1 and LY294002 inhibited p-BAX^Ser184^ expression, while 666-15 had no effect on p-BAX^Ser184^ expression (Fig. 3F). Moreover, 666-15 also had no significant influence in the expression of s-COL11A1 and L-COL11A1 (Fig. 3F). Overexpression of BCL-2 prevented mitochondrial permeability transition pore (MPTP) opening by binding BAX to inhibit apoptotic process switching. p-BAX^Ser184^ cannot insert itself into the mitochondrial membrane to repress MPTP formation. Therefore, we suggest that the COL11A1/Akt/CREB axis inhibits cancer cell apoptosis by promoting BCL-2 and p-BAX^Ser184^ expression and reducing BAX content. In addition, we utilized immunofluorescence to determine the mitochondrial expression and localization of BCL-2 and BAX in BxPC-3 cells in response to different treatments. In accordance with the above experimental findings, COL11A1 decreased expression of BAX and inhibited the binding of BAX to mitochondria but had the opposite effects on BCL-2. siCOL11A1 and LY294002 weakened the function of COL11A1 in regulating the expression and location of BAX and BCL-2 (Fig. 4A). Fluorescence intensity was subsequently determined and normalized against the cellular background fluorescence intensity. Levels of BAX and BCL-2 were calculated as a ratio and compared to the control group (Fig. 4B). These results validated that the COL11A1/Akt/CREB axis exerts antiapoptotic effects on cancer cells and protects tumors from GEM-induced apoptotic cell death by modulating the function of the BAX/BCL-2 signaling node.

### COL11A1 and Akt induce disruption of the mitochondrial transmembrane potential

**Figure 5:**
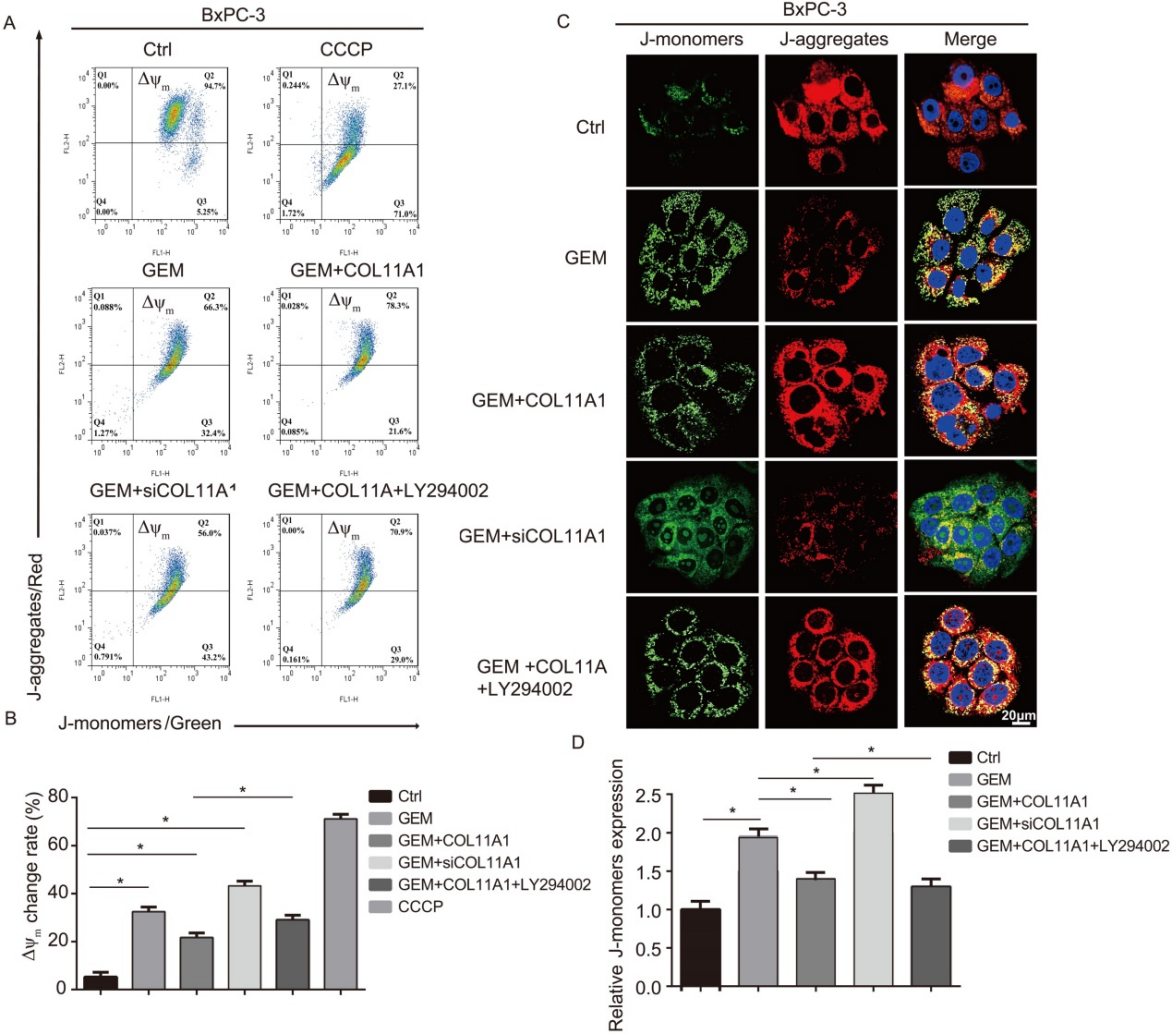
** COL11A1/Akt inhibits the decrease in mitochondrial transmembrane potential.** (A) The results of flow cytometry analyses to determine the Δψ_m_ in BxPC-3 cell after treatment with GEM, COL11A1, siCOL11A1 and/or LY294002 and stained with JC-1 are shown. (B) The relative change in Δψ_m_ was calculated as a ratio compared to the control. Data represent the mean ± SD. (C) BxPC-3 cell treated with GEM, COL11A1, siCOL11A1 and/or LY294002 and stained with JC-1 (the nuclei were stained with DAPI.) were analyzed by confocal microscopy. Confocal images were taken using a 100× objective. (D) The fluorescence intensity was determined and normalized to the cellular background fluorescence. The proportion of J-monomers was calculated as a ratio compared to the number of J-monomers in the control. Data represent the mean ± SD (n=3, *p<0.05).

Mitochondrial apoptosis pathways are the most profound pathways associated with cancer cell resistance. Therefore, we investigated Δψ_m_ following JC-1 staining by flow cytometry and confocal microscopy. When Δψ_m_ is low (during initiation of cellular apoptosis), JC-1 is present as a monomer, called a J-monomer, and shows green fluorescence; when Δψ_m_ is high (when cells are in a normal state), JC-1 forms a polymer called a J-aggregate, which shows red fluorescence. Flow cytometry results showed that in cells treated with GEM, COL11A1, siCOL11A1 and LY294002, COL11A1 treatment suppressed the decrease in Δψ_m_ caused by GEM, but siCOL11A1 and LY294002 promoted this decrease in Δψ_m_. The control group was used as a negative control, and a carbonyl cyanide m-chlorophenylhydrazone (CCCP)-treated group served as a positive control (Fig. 5A and B). Results of confocal imaging indicated that COL11A1 increased the number of J-aggregates and decreased the number of J-monomers compared to those in the GEM-treated group; that is, red fluorescence was enhanced, and green fluorescence was decreased. COL11A1 depletion had the opposite effect. Furthermore, LY294002 significantly inhibited the effect of COL11A1 in suppressing the reduction in Δψ_m_ (Fig. 5C). Fluorescence intensity was determined and normalized against cellular background fluorescence. J-monomer levels were calculated as a ratio compared to controls (Fig. 5D). Therefore, COL11A1 inhibited the decrease in Δψ_m_ through activating Akt. The above results further suggested that COL11A1 inhibits GEM-induced apoptosis.

### The functions of COL11A1/Akt in molecular signaling in the mitochondria-mediated apoptotic program

**Figure 6:**
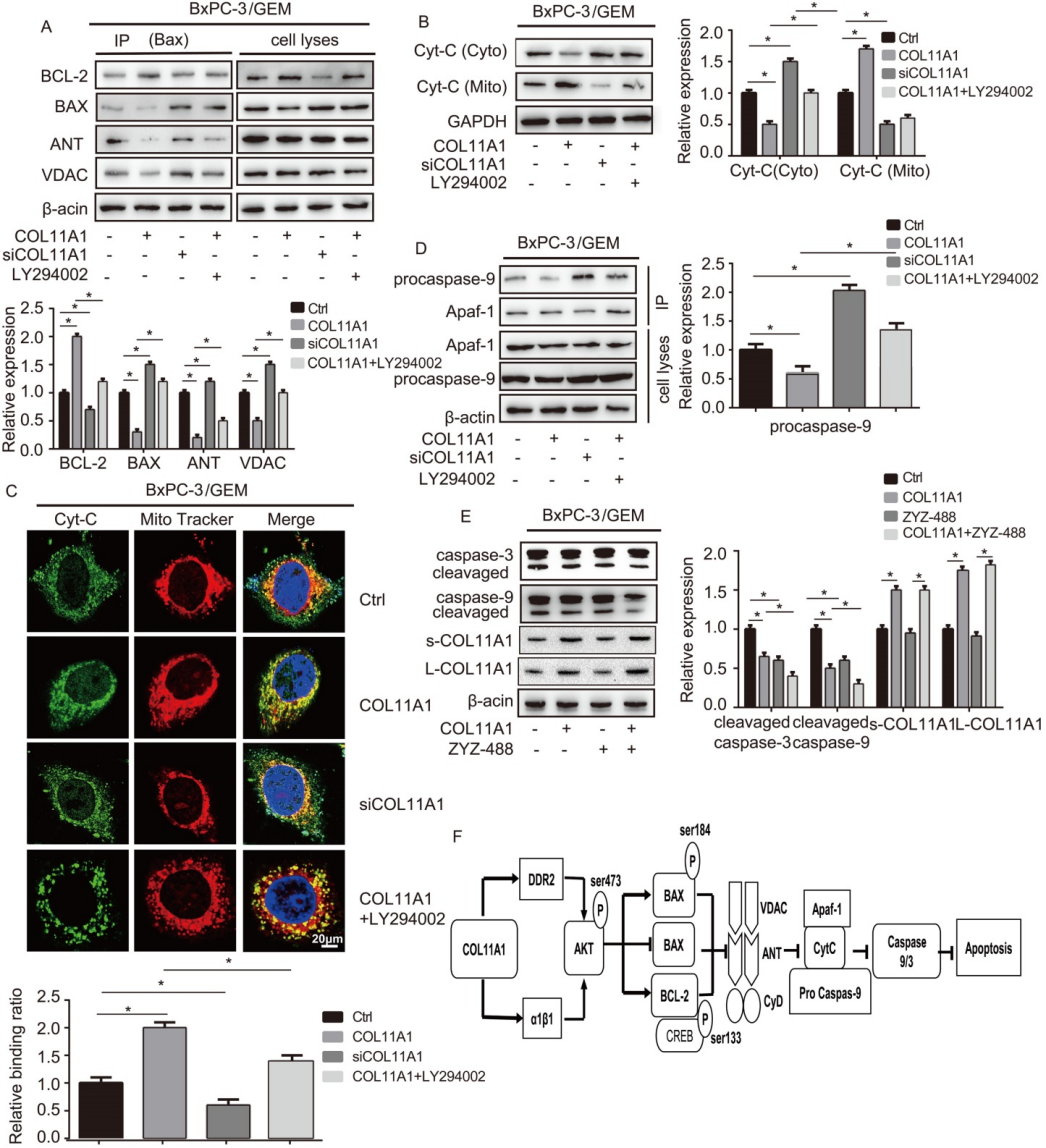
** COL11A1/Akt modulates molecular signaling in mitochondria-mediated apoptosis.** (A) After BxPC-3 cell treated with GEM were stimulated with COL11A1, siCOL11A1 and/or LY294002, the cell lysates were immunoprecipitated with anti-BAX antibody, and immunoblotting were performed with antibodies against BCL-2, BAX, ANT and VDAC. (B) Western blotting analysis of Cyt-C expression in the cytoplasm and mitochondria of BxPC-3 cell treated with COL11A1 or siCOL11A1 and/or LY294002 were carried out. (C) The results of immunofluorescence analysis of Cyt-C expression in BxPC-3 cell following different treatments are shown. Mitochondria were visualized with MitoTracker (red); Cyt-C were visualized with Alexa Fluor 488 (green), and nuclei were stained with DAPI. Confocal images were taken using a 200× objective. The fluorescence intensity was determined and normalized to the cellular background fluorescence. The levels of Cyt-C were calculated as a ratio compared to those in the controls. Data represent the mean ± SD. (D) BxPC-3 cell were treated with COL11A1, siCOL11A1 and LY294002. Cell lysates were immunoprecipitated with anti-Apaf-1 antibody, and immunoblotting was performed with anti-procaspase-9. (E) The cleaved caspase-3/9 protein, L-COL11A1 and s-COL11A1 levels of BxPC-3 cell under different conditions were detected by western blotting. (F) A schematic diagram shows the functions that are altered due to COL11A1.

Apoptosis is related to Δψ_m_, and changes in Δψ_m_ are related to the MPTP, which is formed by the BAX, ANT, VDAC and CyD complex and inserted into the mitochondrial membrane. Therefore, we assessed the relationship between COL11A1 and MPTP. Studies have demonstrated that BAX modulates Disparkin-mediated transport through the MPTP by binding ANT, VDAC, BCL-2 and BAX to generate MPTP complexes in the mitochondrial membrane 53, 54. To examine the regulatory effect of COL11A1 on BAX and the ANT, VDAC, BCL-2, and BAX complex, BxPC-3 cells treated with GEM were stimulated with COL11A1, siCOL11A1 and/or LY294002. Then, cell lysates were immunoprecipitated using anti-BAX antibody, the results of which demonstrated that COL11A1 increased the formation of the BAX and BCL-2 complex but decreased formation of the complex between BAX, ANT and VDAC. siCOL11A1 and LY294002 exerted the opposite effect (Fig. 6A). The above results indicate that COL11A1 reduces the number of MPTP openings to inhibit the decrease in Δψ_m_, which inhibits apoptosis. Disparkin induces Cyt-C release through the MPTP from the mitochondria into the cytoplasm. Therefore, we examined the expression of Cyt-C in both mitochondria and the cytoplasm of BxPC-3 cells following different treatments by western blotting. We found that COL11A1 increased protein expression of Cyt-C in the mitochondria and decreased Cyt-C expression in the cytoplasm compared to its expression in the group treated with GEM alone. However, siCOL11A1 had the opposite effects, and LY294002 suppressed the effect of COL11A1 on Cyt-C expression (Fig. 6B). We further confirmed the effect of COL11A1 on the distribution of Cyt-C using immunofluorescence. Mitochondria were visualized using MitoTracker (red), and Cyt-C was visualized using Alexa Fluor 488 (green). Results were consistent with those shown in Fig. 6B (Fig. 6C). Cyt-C in the cytoplasm forms a complex with Apaf-1 and procaspase-9, initiating the apoptosis program, so we further detected expression of the complex through immunoprecipitation. Results revealed that COL11A1 inhibited formation of the complex, while siCOL11A1 and LY294002 promoted complex formation (Fig. 6D). We also found that ZYZ-488 (an inhibitor of Apaf-1) strengthened the effect of COL11A1 on cleaved caspase-9/3 expression, while showed no significant influence in the expression of s-COL11A1 and L-COL11A1 (Fig. 6E). A schematic diagram showing the functions altered by COLL1A1 is presented in Fig. 6F. Consequently, these findings indicate that the COL11A1/Akt axis functions to suppress the mitochondria-mediated apoptotic program in pancreatic cancer cells through molecular signaling.

## Discussion

Collagen contributes to the composition of the tumor microenvironment, especially in PDAC, which is characterized by obvious desmoplastic stromal reactions. Targeting the cancer-stroma interaction is thought to represent a new therapeutic approach for pancreatic cancer 55. In the present study, we utilized multiple experiments to explore COL11A1 function in PDAC development. We demonstrated that COL11A1 activates Akt signaling to enhance the proliferation of pancreatic cancer cells and their ability to evade apoptosis. Consistent with previous clinical studies 24-26, we show that COL11A1 facilitates tumor progression in PDAC. Over the years, fibrillar collagens I, II, III, and V have been reported to be involved in tumor development, and many elaborate mechanistic studies have been presented 56, 57. However, the specific mechanism by which collagen induces the pathogenesis of PDAC remains unknown. Normally, collagen XI/COL11A1 is scarce in most tissues under physiological conditions. Therefore, alterations in its expression associated with cancer might be characteristic of neoplastic transformation and serve as an indicator of such transformation 58. Structurally, collagen XI α1 and α2, but not collagen α3, resemble type V collagen, and their specific procollagenases are cleaved, prompting their secretion into the ECM where they engage in molecular signaling 59. Thus, COL11A1 is the active subunit of collagen XI. Herein, we showed that COL11A1 is conducted by integrin α1β1 and DDR2 to mediate cancer cell proliferation and activate Akt^Ser473^ signal transduction. Collagen binds the integrin headpiece, which consists of the β-propeller/I domain of the α-subunit and the N-terminal domain of the β-subunit, at collagen-binding sites (CBSs). The CBS of DDR2 has three spatially adjacent surface-exposed loops in the discoidin domain, which are arranged such that they recognize and bind specific collagen. DDR2 affects integrin α1β1-collagen binding in combination or independently to modulate their effects 27. We found that integrin α1β1 and DDR2 mediate COL11A1-induced p-Akt^s437^ activation during pancreatic cancer development, suggesting that blocking integrin α1β1 or DDR2 to inhibit the functions of COL11A1 is specific but not exclusive. The specific collagenous amino acid sequences recognized by integrin α1 domains include GFOGER, GLOGER, GASGER, GROGER and GVMGFO motifs. However, the motifs that recognize COL11A1-integrin α1β1 binding are still unknown. In general, DDR2 distinctively binds the collagen GVMGFO motif, enhancing the affinity of integrinα1 for its collagen ligands, GMOGER, GAOGER and GLOGEN 60. However, in present study, we found there was no synergistic effect between integrin α1 and DDR2 in regulating pancreatic cancer cell function. Consequently, we propose that COL11A1 does not contain GMOGER, GAOGER and GLOGEN ligands domains. Thus, GFOGER, GROGER, GASGER and GVMGFO sequences might be the specific motifs that target the function of COL11A1 in modulating PDAC tumorigenesis.

In the current work, we further revealed that COL11A1 inhibits apoptosis in pancreatic cancer cells by activating Akt/CREB/BCL-2 signaling. Akt/PKB plays important roles in the mechanism by which responses to extracellular stimuli regulate cell survival and proliferation. Some transcription factors generated by Akt/PKB, including the forkhead family, Mdm2, CREB, YAP and NF-κB, control cell survival. Among these proteins, cyclic AMP-response element binding protein (CREB)/integrin and CREB-binding protein (CBP) in particular regulate ECM-mediated cell growth and apoptosis during carcinogenesis 61. Therefore, COL11A1 is thought to preferentially phosphorylate Akt^Ser473^/CREB^Ser133^ to promote cancer cell survival in PDAC. Furthermore, Akt^Ser473^ and CREB^Ser133^ play a vital role in controlling the ECM-associated proliferation of pancreatic cancer cells under direct regulation of COL11A1. Therefore, the COL11A1/Akt^Ser473^/CREB^Ser133^ cascade might represent an effective target for interfering with the tumor-permissive ECM in PDAC. Next, we observed that Akt^Ser473^/CREB^Ser133^ activation by COL11A1 modulates the coordinated functions of BAX/BCL-2 in decreasing the apoptotic program in pancreatic cancer cells. As apoptosis is associated with several cellular activities and has profound effects on the progression of malignancy, it has been suggested as a target for the treatment of various cancers 32. Mitochondrial apoptosis is involved in some important factors, including the MPTP, Δψ_m_ and mitochondrial outer membrane permeabilization (MOMP). Members of the BCL-2 family (antiapoptotic and proapoptotic proteins) converge at the mitochondria to regulate the molecular apoptotic pathway. Proapoptotic proteins promote MPTP formation and MOMP and reduce Δψ_m_ to enhance the release of Cyt-C, which irreversibly initiates apoptosis, while antiapoptotic proteins prevent this function. Among members of the basic circuitry for evasion of the apoptotic process in cancer, BAX and BCL-2 are tightly coregulated, inhibiting mitochondrial function 32. In the present study, the mechanism by which COL11A1/Akt^Ser473^/CREB^Ser133^ regulate antiapoptotic processes was determined. We found that inactivation of the proapoptotic protein BAX alters its mitochondrial/cytosolic distribution and transcriptionally increases expression of the prosurvival protein BCL-2, which translocates into the mitochondrial outer membrane in pancreatic cancer cells. The consequent increased BCL-2/BAX ratio results in cancer cell evasion of apoptosis by inhibiting activity of the MPTP and MOMP and increasing Δψ_m_. We observed that COL11A1/Akt^Ser473^/CREB^Ser133^ promotes BCL-2 levels and p-BAX^Ser184^ activity while decreasing BAX expression, which suppresses formation of the BAX/BAX homodimer, BAX/VDAC heterodimer and BAX/ANT/VDAC complex, deactivating MPTP function, increasing Δψ_m_ and decreasing MOMP, blocking leakage of Cyt-C into the cytosol and the combination of the Apaf-1/procaspase-9/Cyt-C complex. Therefore, the cancer cell apoptosis program is sequentially impeded. Importantly, BAX activity and transcription have been shown to be depressed by direct Akt activation, as well as by BCL-2 expression 62. Thus, the present findings indicate that BAX acts as a fundamental promoter of the molecular cascade by which COL11A1 mediates antiapoptotic events in pancreatic cancer cells. Therefore, we demonstrated that a therapeutic method capable of depressing the Akt/BAX interaction should promote efficient apoptosis and limit proliferation in PDAC with high COL11A1 expression.

Moreover, we identified that COL11A1/Akt signaling decreased the proapoptotic activity of GEM in pancreatic cancer cells, which, in turn, attenuated its anticancer properties. GEM inhibits DNA synthesis to induce nuclear apoptosis and cell cycle arrest during S phase 39. A better understanding of the mechanisms of GEM indicate that it activates HIPK2, p38 MAPK, DYRK2 and PKC kinases, which are involved in p53^Ser46^ phosphorylation, executing cell apoptosis in response to DNA damage in various cancers 63. However, the effect of GEM is primarily implemented in a p53-independent manner, as suggested by the finding that the p53 gene is mutated or lost in 75% of pancreatic cancer cells 64. Therefore, it is important to adequately investigate the mechanism of GEM cytotoxicity to potentiate its activity and prevent chemoresistance in PDAC. Activation of Akt leads to the phosphorylation of BAX, increasing drug resistance in a p53-independent manner in several cancers 65, 66. p-Akt^Ser473^ is overexpressed in PDAC patients. Additionally, recent studies have indicated that targeting Akt activity overcomes GEM resistance in some cancers 67-69. Notably, we selected a p53-mutant pancreatic cancer cell line (BxPC-3) to assess modulation of Akt/PI3K-dependent activity of GEM by COL11A1. GEM decreased Akt activity, causing mitochondrial apoptosis in pancreatic cancer cells; however, COL11A1 impaired this effect. In addition, some studies have reported that p-Akt^Ser473^ reduces BAX expression by regulating YAP/P73 signaling in p53-mutant cells 41. Therefore, the COL11A1/Akt axis was shown to be a core signaling pathway associated with PDAC chemoresistance. Hence, this method of manipulation might provide new opportunities for protection from GEM resistance in PDAC through implementation of COL11A1 inhibitors. Consequently, we propose that the COL11A1/Akt signaling pathway can be utilized to predict the therapeutic efficacy of GEM and as a novel agent to synergize with the anticancer activity of GEM.

It has been reported that inhibition of Akt with SC66 reduces expression levels of COL11A1 in ovarian cancer cells 70, indicating that Akt signaling might induce expression of COL11A1. Herein, we showed that the fragment of COL11A1 protein promotes the activation of Akt signaling; hence, we hypothesized that activation of Akt signaling in turn promotes expression of full length COL11A1. As expected, inhibition of Akt signaling with LY294002 decreased the expression levels of COL11A1 induced by COL11A1 fragment in both supernatant medium and total cell lysate. Furthermore, downregulation of full length COL11A1 weakened the activation of Akt signaling, indicating that there might be positive feedback between Akt signaling and COL11A1. In subsequent studies, we intend to introduce the plasmid to upregulate COL11A1 expression to further clarify COL11A1's role and mechanism in PDAC progression.

In conclusion, COL11A1 mediates apoptosis inhibition and chemoresistance in pancreatic cancer cells by activating the Akt/CREB/BCL-2/BAX signaling pathway. COL11A1 could be developed as a distinctive indicator of prognosis and represents an attractive therapeutic target for PDAC.

## Supplementary Material

Supplementary figure S1.

## Figures and Tables

**Table 1:** Sequences of the siRNAs used

Gene	siRNA sequences (5' to 3', forward to reverse)
siCOL11A1-1	sense: 5'- CUCCAGUUGAUGUACUAAATT -3'
	antisense: 5' - UUUAGUACAUCAACUGGAGTT-3'
siCOL11A1-2	sense: 5'- CCAGAGGAUAUAAUCGAAUTT-3'
	antisense: 5'- AUUCGAUUAUAUCCUCUGGTT-3'
siα1-1	sense: 5'- GCCCUUAUAUGCCUAUAGATT-3'
	antisense: 5'- UCUAUAGGCAUAUAAGGGCTT-3'
siα1-2	sense: 5'- GCUGCUGCGUAUCAUUCAATT-3'
	antisense: 5'- UUGAAUGAUACGCAGCAGCTT-3'
siβ1-1	sense: 5'- GAACAGAUCUGAUGAAUGATT-3'
	antisense: 5'- UCAUUCAUCAGAUCUGUUCTT-3'
siβ1-2	sense: 5'- GUGGUUUCGAUGCCAUCAUTT-3'
	antisense: 5'- AUGAUGGCAUCGAAACCACTT-3'
siDDR2-1	sense: 5'-CCAGAUUUGUCCGGUUCAUTT-3'
	antisense: 5'- AUGAACCGGACAAAUCUGGTT-3'
siDDR2-1	sense: 5'- GACCGCAUCAGGAAUUUCATT -3'
	antisense: 5'- UGAAAUUCCUGAUGCGGUCTT-3'

**Table 2:** Primer sets used for RT-qPCR analysis

Gene	Primer sequences (5' to 3', forward to reverse)
α1	Forward: 5'- CACCTTTCAAACTGAGCCCGCCA-3'
	Reverse: 5'- GCTGCCCAGCGATGTAGAGCACAT-3'
β1	Forward: 5'- TTCAGACTTCCGCATTGGCTTTGG-3'
	Reverse: 5'- TGGGCTGGTGCAGTTTTGTTCAC-3'
DDR2	Forward: 5'- AGGATGATCCCGATTCCCAGA-3'
	Reverse: 5'- GATCCGAGTGTTGCTATCATCAAC-3'
BCL-2	Forward: 5'- TTCTTTGAGTTCGGTGGGGTC -3'
	Reverse: 5'- TGCATATTTGTTTGGGGCAGG -3'
BAX	Forward: 5'- -ATCCAGGATCGAGCAGGGCG-3'
	Reverse: 5'- ACTCGCTCAGCTTCTTGGTG-3'
COL11A1	Forward: 5'- TTGATGTTACCGTTCCGTTATG-3'
	Reverse: 5'-AATCCGAGCCTGCTGAAGAAT-3'
GAPDH	Forward: 5'-TTGTCTCCTGCGACTTCA-3'
	Reverse: 5'-CCACCACCCTGTTACTGTT-3'
