# Supplementary material for: The COL11A1/Akt/CREB signaling axis enables mitochondrial-mediated apoptotic evasion to promote chemoresistance in pancreatic cancer cells through modulating BAX/BCL-2 function
Source: J Cancer. 2021 Jan 1;12(5):1406–20. doi: 10.7150/jca.47032 (PMC7847647; doi:10.7150/jca.47032)

Supplementary Figure 1: (A) The expression level of s-COL11A1 in the four pancreatic cancer cells (BxPC-3, Capan-1, Mia PaCa-2, PANC-1) treated with coating-COL11A1 for 48h were tested by ELISA. (B)The expression level of s-COL11A1 and L-COL11A1 in the four pancreatic cancer cells (BxPC-3,Capan-1,Mia PaCa-2,PANC-1) treated for 0h,12h,24h,48h were detected by western blotting. (C) Western blotting analysis was performed to test the effect of GEM on s-COL11A1, L-COL11A1, integrin α1β1 and DDR2. (D) Knockdown efficiency of siRNA targeting COL11A1, integrin α1β1 and DDR2 were detected by western blotting. Data represent the mean ± SD. ($n = 3$, $^{*}P < 0.05$).

Supplementary Figure 1

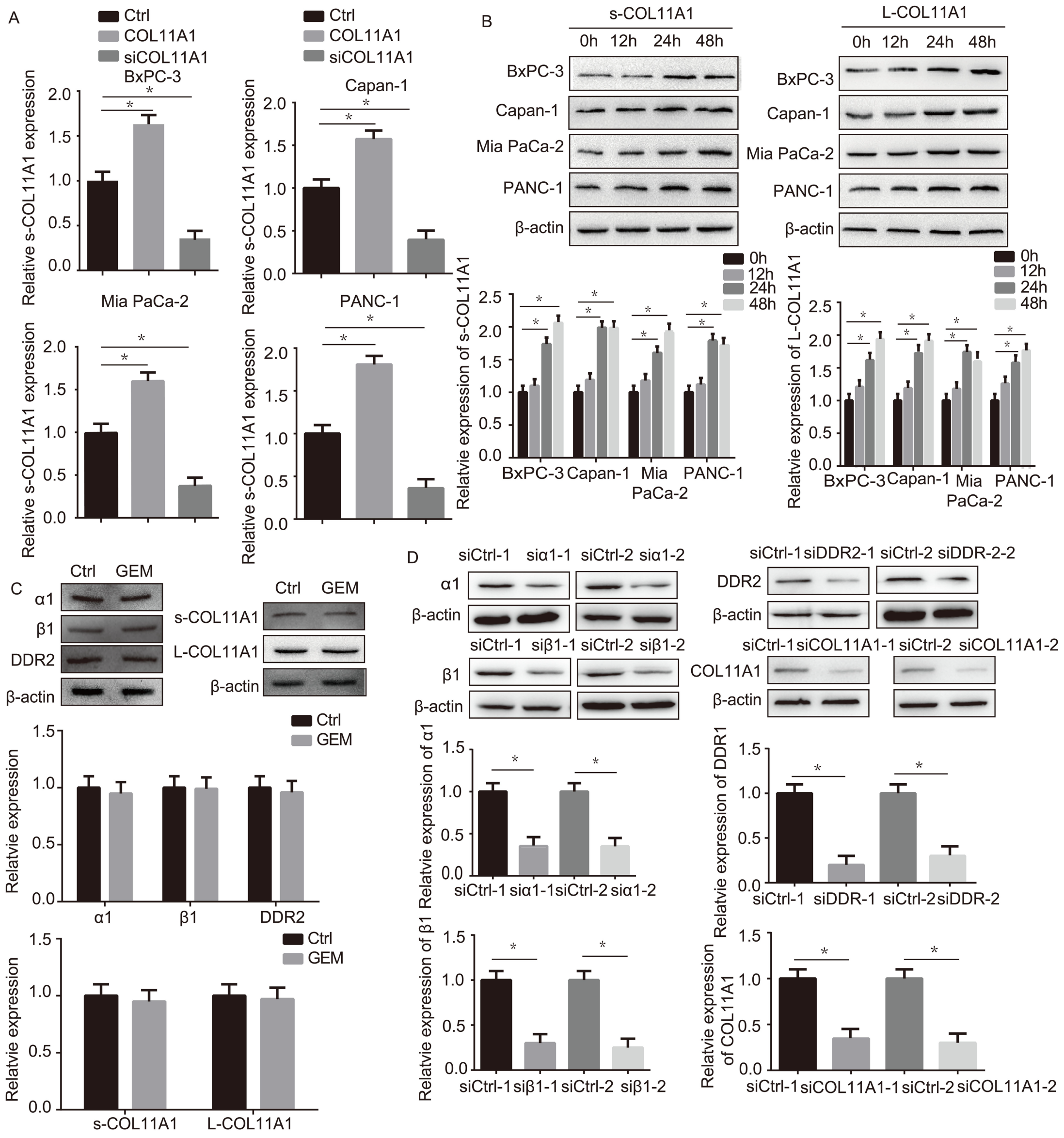

Supplement: Supplementary file 1 — Supplementary figure S1. [file jcav12p1406s1.pdf]
